# Description of an aerodynamic levitation apparatus with applications in Earth sciences

**DOI:** 10.1186/1467-4866-11-4

**Published:** 2010-09-27

**Authors:** Andreas Pack, Katrina Kremer, Nina Albrecht, Klaus Simon, Andreas Kronz

**Affiliations:** 1 Georg-August-Universität, Geowissenschaftliches Zentrum, Goldschmidtstraße 1, D-37077 Göttingen, Germany

## Abstract

**Background:**

In aerodynamic levitation, solids and liquids are floated in a vertical gas stream. In combination with CO_2_-laser heating, containerless melting at high temperature of oxides and silicates is possible. We apply aerodynamic levitation to bulk rocks in preparation for microchemical analyses, and for evaporation and reduction experiments.

**Results:**

Liquid silicate droplets (~2 mm) were maintained stable in levitation using a nozzle with a 0.8 mm bore and an opening angle of 60°. The gas flow was ~250 ml min^-1^. Rock powders were melted and homogenized for microchemcial analyses. Laser melting produced chemically homogeneous glass spheres. Only highly (e.g. H_2_O) and moderately volatile components (Na, K) were partially lost. The composition of evaporated materials was determined by directly combining levitation and inductively coupled plasma mass spectrometry. It is shown that the evaporated material is composed of Na > K >> Si. Levitation of metal oxide-rich material in a mixture of H_2 _and Ar resulted in the exsolution of liquid metal.

**Conclusions:**

Levitation melting is a rapid technique or  for the preparation of bulk rock powders for major, minor and trace element analysis. With exception of moderately volatile elements Na and K, bulk rock analyses can be performed with an uncertainty of ± 5% relative. The technique has great potential for the quantitative determination of evaporated materials from silicate melts. Reduction of oxides to metal is a means for the extraction and analysis of siderophile elements from silicates and can be used to better understand the origin of chondritic metal.

## Background

### Aerodynamic levitation

The term aerodynamic levitation is used for a technique, in which solids or liquids are freely floated on top of a vertical gas stream. With this technique, samples are not in contact with any container material. Therefore, aerodynamic levitation allows the conduction of high-temperature experiments while avoiding problems related to the chemical interaction between sample and container walls (e.g., corrosion of oxide crucibles by silicate melts [1,2], gain or loss of siderophile elements and Fe in Pt crucibles, [3,4]).

Oxides and silicates can be heated and melted with a CO_2 _gas laser [5], a mirror furnace [6], or a solar furnace [7], while they are floating on top of the gas stream. Depending on the supplied energy, temperatures >3000°C can be reached [8].

Aerodynamic levitation has been used for studies of the physical properties of solids [5] and liquids [9-13] at high temperatures. It has also been used to investigate the crystallization behavior of oxides [14] and silicates [15-17]. Due to the absence of heterogeneous nucleation sites, aerodynamic levitation was also used for the preparation of glasses from substances that otherwise crystallize during cooling [18-20].

In this contribution, we describe an aerodynamic levitation device and demonstrate advantages and limitations of aerodynamic levitation in combination with laser heating for a) sample preparation for bulk rock chemical analyses, b) high-temperature evaporation experiments and c) reduction experiments.

### Application fields of aerodynamic levitation in Geosciences

#### Sample preparation for bulk rock chemical analyses

Bulk rock major, minor and trace element analyses are an integral part of modern geological studies [21]. Conventionally, bulk rock analyses are obtained by X-ray fluorescence on fused glass disks, by mass or by optical spectroscopy of dissolved materials, or by instrumental neutron activation analysis. Recently, major, minor and trace element concentrations have been determined on fused bulk rock samples by laser ablation inductively coupled mass spectrometry (LA-ICPMS) [22-26]. Bulk rock glasses were prepared by means of fusion with Li-borate flux in a Pt crucible [22,25] or by melting on an electrically heated Ir strip [23,27]. The latter technique requires less material ( < 50 mg), but results in the loss of some volatile elements from the sample.

Pack et al. [28], Pack [29] and Patzer et al. [30] used aerodynamic levitation in conjunction with CO_2 _laser melting as preparation technique for analyses of Ca, Y, REEs, Zr, and Hf in bulk chondrites and achondrites. The analyses were conducted using LA-ICPMS. The concentration of Ca, which was used as internal standard, was determined by electron microprobe analyses (EPMA). Only about 10 mg of sample material was required. In Pack et al. [28], 17 chondrites were analyzed for Y and Ho at concentration levels of ~2.4 μg g^-1 ^and ~0.09 μg g^-1^. Small variations ( ± 5%) of the Y/Ho ratios were related to fractional condensation processes in the solar nebula. Pack [29] demonstrated that REEs can be determined with a precision better than ± 5% on fused bulk meteorites. It was demonstrated that bulk chondrites do not have unfractionated REE abundances. Patzer et al. [30] showed that precise and accurate bulk rock Zr/Hf ratios can be obtained by levitation melting and subsequent LA-ICPMS analysis. Fractionations among refractory lithophile elements were related to processes in the solar nebula 4.6 Ga ago.

Details of the levitation apparatus, however, were not described by Pack et al. [28], Pack [29] and Patzer et al. [30] and will be presented in this contribution. It will also be demonstrated that laser-assisted melting of ~10 mg rock powders is suitable not only for refractory trace element (Y, REE, Zr, Hf), but also for bulk major and minor element analyses.

#### Evaporation experiments

##### Alkali evaporation during chondrule melting

**Figure 1:**
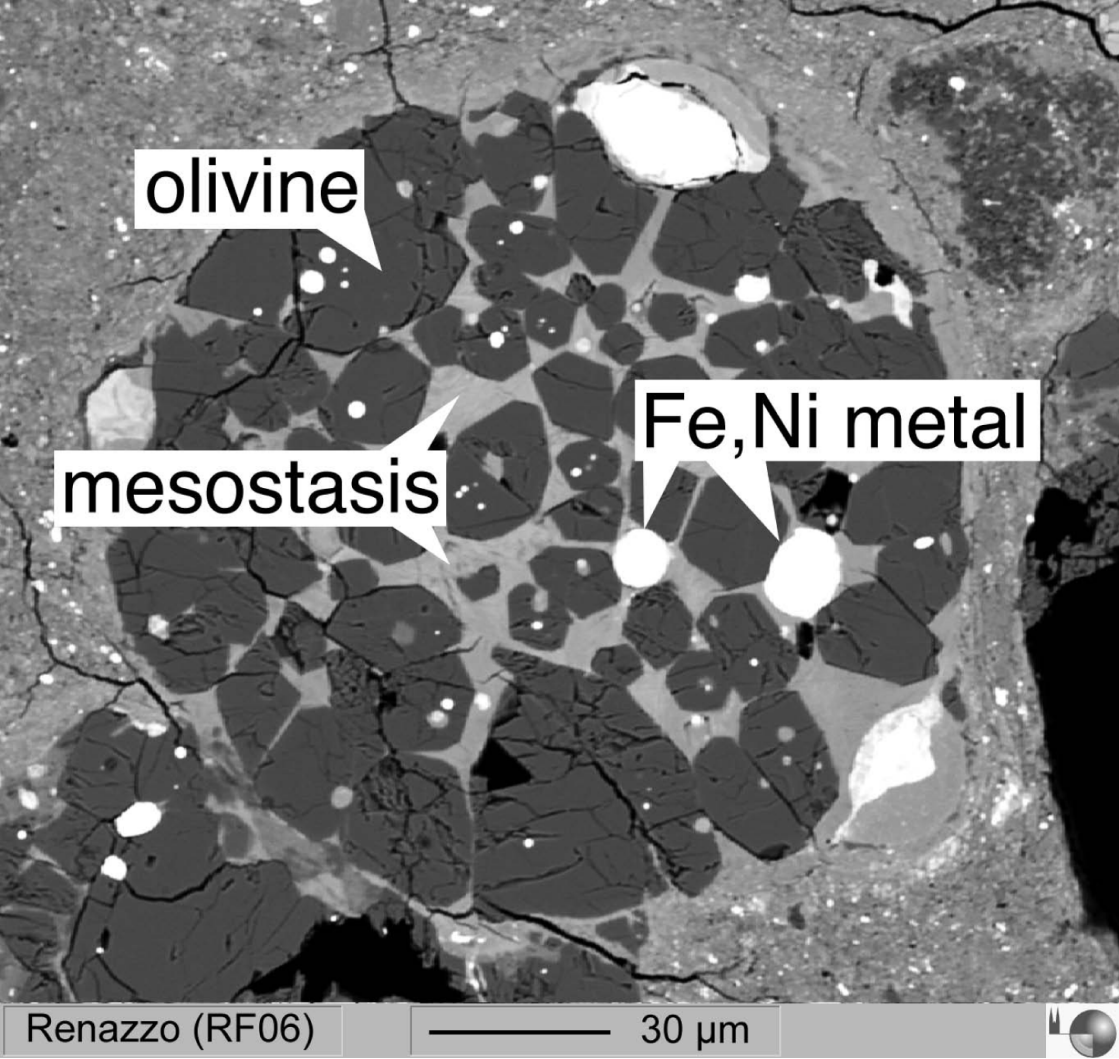
**Back scattered electron image of a chondrule from the Renazzo (CR2) carbonacenous chondrite [modified after **[37]**]**. The chondrule contains olivine, metal and intersitial mesostasis.

Alkalis belong to the group of moderately volatile elements [31]. In cosmochemical context, evaporation of alkalis provides important insights into the conditions (pressure, oxygen fugacity, duration) of chondrule melting in the protoplanetary disk [32,33]. Chondrules are ~0.1 - 1 mm sized silicate spheres (Fig. 1) that formed by a brief, but intense melting event in the solar nebula [34]. They are major constituents of chondritic meteorites. In a low-pressure (10^-6 ^to 10^-4 ^bar), H_2_-rich nebular gas, alkalis are expected to evaporate within minutes from the melt. However, it was shown by Borisov et al. [33] and Alexander et al. [32] that chondrules did not lose alkalis during melting. Melting of chondrules in a non-canonical high-pressure nebular gas [35] may explain the observed absence of alkali evaporation.

We will demonstrate that evaporation of alkalis from levitated molten silicates can be monitored with high time resolution. The applicability of the experimental procedure with respect to the problem of alkali-retention in chondrules will be discussed.

#### In-situ reduction of silicate melts

Chondrites and chondrules contain metal that did not form by direct condensation from the solar nebula. Instead, it has been suggested that metal in chondrites formed by reduction from oxides during the chondrule melting event [36]. Some chondrules contain Fe, Ni metal blebs that may have formed by reduction during the brief chondrule melting event (Fig. 1, modified after [37]). In this contribution, we demonstrate that Fe, Co, Ni and likely other and more siderophile elements can be transferred from the oxidized form into liquid metal during levitation.

The usability of the technique for the analysis of siderophile trace elements in rocks will also be discussed.

## Results

### The aerodynamic levitation apparatus and sample preparation

**Figure 2:**
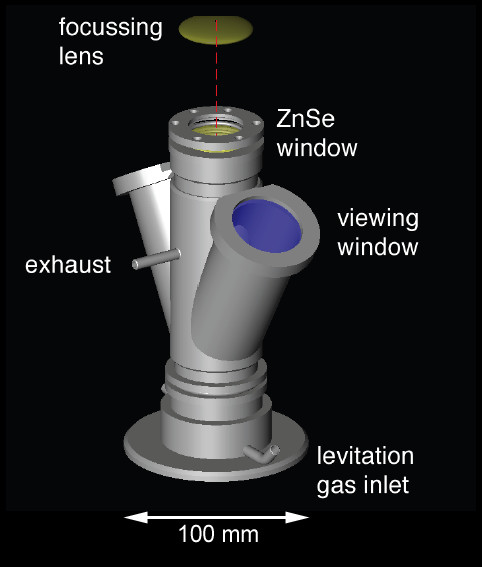
**Sketch illustrating the levitation apparatus**. The levitation nozzle was placed inside the sample chamber. The chamber comprised sapphire windows for viewing and illumination and a ZnSe window for the laser beam. The levitation gas could be conducted via the exhaust to the source of an ICPMS for chemical analyses.

The levitation device comprised an infrared (IR) CO_2 _laser heat source, a levitation chamber with levitation nozzle and a levitation gas supply (Fig. 2).

**Figure 3:**
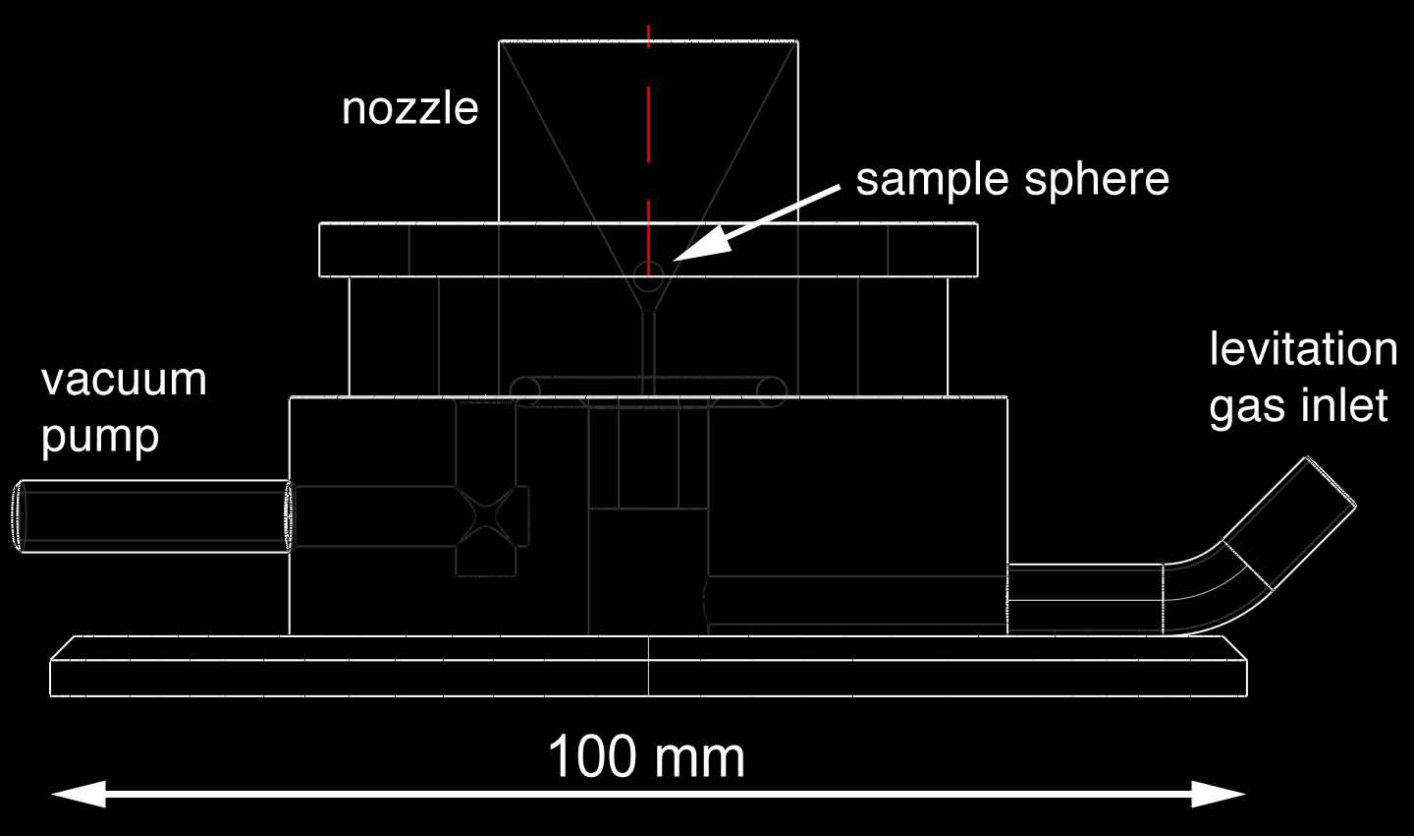
**Cross section through the lower part of the levitation apparatus (sample chamber removed)**. The levitation nozzle comprised a 0.8 mm bore and an opening angle of 60°.

We used a SYNRAD 50 W CO_2 _laser (λ = 10.4 μm) as heat source. The laser was focused to a beam diameter of ~1.5 - 2 mm by means of a ZnSe lens (ƒ = 125 mm). The laser energy output could continuously be varied between 0 and 95%. The levitation chamber hosted the levitation nozzle. The chamber was used when conducting experiments in controlled atmospheres (e.g., under reducing conditions) were carried out (or the chamber was used during conducting experiments in controlled atmospheres). Two sapphire windows were used for video monitoring and for illumination. The top of the chamber was covered with an IR transparent ZnSe window. The levitation nozzle had an opening angle of 60° and a 0.8 mm bore (Fig. 3). It was made of aluminum. The levitation gas was regulated with a computer-controlled VÖGTLIN red-y mass flow controller (0 - 1000 mL min^-1^).

Aerodynamic levitation experiments required solid, nearly spherical samples as starting material. One way of preparing such a starting sample was to briefly fire the laser (defocused to ~2 mm, ~1 s) directly into a pot with the sample powder. The powder melted and solidified as nearly spherical glassy droplet. The glass beads could be placed upside down, i.e. with the molten surface directed toward the levitation gas stream in the levitation nozzle. Alternatively, the sample powder (~10 - 20 mg) could be placed in a spectroscopy-grade graphite crucible (~5 mm inner diameter, ~4 mm depth) and briefly melted into a glass bead. The resultant bead could be placed in the nozzle for levitation.

**Figure 4:**
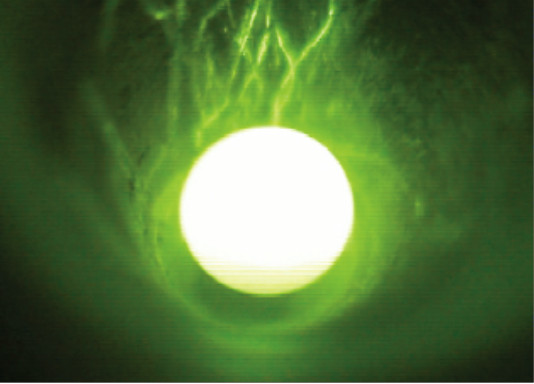
**Video image showing a droplet of liquid basalt BCR-2 during levitation**. The sample was heated from the top using a CO_2_-laser. The diameter of the sphere was ~2 mm.

Stable levitation was a prerequisite for sample homogenization for chemical analyses as well as for evaporation and reduction experiments. The nozzle with the 60° opening angle and 0.8 mm bore allowed to keep molten silicates in a stable position for up to >1 h (Fig. 4). We tested also a nozzle with a 0.8 mm bore, but a wider opening angle of 120°. However, we could not keep the spheres in stable positions with this nozzle. The gas flow was set to 250 - 300 mL min^-1 ^for silicate spheres with ~2 mm diameter. Smaller as well as larger spheres failed to float stably. The distance between sphere and nozzle was ~1 mm. The aluminum metal nozzle did not reach temperatures exceeding ~100°C even in the long-run experiments at full laser power.

### Sample preparation for bulk rock chemical analyses

In order to demonstrate if aerodynamic levitation melting is suitable for major and minor element bulk rock analyses, we prepared glassy spheres of 7 United States Geological Survey (USGS) standard rock powders: BIR-1 (basalt, 48.0 wt.% SiO_2_), BHVO-2 (basalt, 49.9 wt.% SiO_2_), W-2 (diabase, 52.7 wt.% SiO_2_), BCR-2 (basalt, 54.1 wt.% SiO_2_), AGV-2 (andesite, 59.3 wt.% SiO_2_), GSP-2 (granodiorite, 66.6 wt.% SiO_2_), and RGM-1 (rhyolite, 73.4 wt.% SiO_2_). Bulk compositions (recommended values) from the GeoReM database [38] were taken.

In preparation for levitation, the rock standard powders were pre-fused in small graphite crucibles. A new crucible was machined for each sample in order to avoid any cross contamination. The pre-fused sample beads were placed upside down, i.e. with the glassy surface in the levitation nozzle, levitated and melted between 1 and 4 times for ~5 s each in an Ar atmosphere (i.e. we used Ar as levitating gas). Argon gas was used because we wanted to avoid changes in oxidation state of the samples. High concentrations of ferric Fe in chondritic samples that were levitated in air led to formation of abundant <10 μm sized quenched spinel crystals [28-30].

The attached video system was used to monitor the fusion process. Remaining crystals were visible as bright spots in the slowly rotating and convecting melts. Only samples with no visible crystals left were used for the chemical analyses. The disappearance of crystals indicated that the liquidus temperature was reached. Samples were fused at temperatures only little above their liquidus in order to minimize evaporation.

We quenched the samples by switching off the laser. The cooling rates for aerodynamic levitation experiments with 1 mm forsterite spheres were reported to be in the range of 700 °C s^-1 ^[19]. As a result of the high cooling rates and absence of sites for heterogeneous nucleation, all silicate rock standards solidified as glass.

The spheres were removed from the nozzle by means of a pair of tweezers after they cooled down in the Ar gas stream. They were stored in small glass vials. We embedded the glass spheres into resin and prepared polished 1-inch sections. The sections were carbon coated before EPMA. One section contained up to >40 spheres.

We used a JEOL 8900R electron microprobe for major and minor element (Si, Ti, Al, Mg, Fe, Mn, Na, K; Kα lines) chemical analyses of the USGS rock standard glasses. We used a defocused beam (25 μm) with acceleration voltage of 15 kV and an electron current of 15 nA. The microprobe was calibrated with a set of natural and synthetic silicates and oxides. The calibration was complemented by analyses of reference glasses KL2, GOR128, GOR132, ML3B, T1, StHs6/80 and ATHO [27].

We analyzed 10 randomly selected spots on each of the USGS rock standard glasses. The results of EPMA of the investigated 7 USGS rock standards are listed in Table 1. The internal heterogeneity (major and minor elements, EPMA spots) of the spheres was in all cases <5% (1σ SE, N = 10).

**Table 1:** Results of EPMA measurements of levitated and fused USGS rock standards.

Sample	Times fused	SiO_2_	TiO_2_	Al_2_O_3_	MnO	Fe_2_O_3_	MgO	CaO	Na_2_O	K_2_O	Total
BIR-2	1×	46.5	0.91	15.0	0.18	13.0	9.47	12.91	1.81	0.03	99.86
	2×	47.7	0.95	15.6	0.17	11.3	9.62	13.10	1.48	0.02	99.94
	3×	47.5	0.95	15.8	0.18	11.1	9.69	13.23	1.09	0.02	99.63
	4×	47.4	0.95	16.0	0.17	11.2	9.93	13.47	0.57	0.02	99.69
BHVO-2	1×	49.6	2.68	13.5	0.17	12.2	7.25	11.09	2.16	0.48	99.16
	2×	49.7	2.69	13.8	0.16	12.3	7.23	11.27	1.81	0.41	99.25
	3×	49.7	2.82	14.2	0.18	12.2	7.42	11.59	1.22	0.27	99.63
	4×	49.7	2.86	14.3	0.18	12.3	7.50	11.73	1.09	0.25	99.93
AGV-2	1×	60.0	1.04	17.9	0.11	6.8	1.84	5.31	3.36	2.44	98.82
	2×	59.7	1.07	18.4	0.10	6.7	1.86	5.40	3.19	2.22	98.74
	3×	60.4	1.09	18.8	0.10	6.7	1.90	5.48	2.86	2.10	99.40
	4×	57.4	1.25	21.7	0.11	7.1	2.21	6.25	1.79	1.42	99.18
BCR-2	1×	54.7	2.30	13.9	0.18	13.6	3.65	7.12	2.54	1.41	99.36
	2×	54.9	2.30	14.2	0.19	13.8	3.67	7.30	2.10	1.31	99.72
	3×	54.1	2.50	15.2	0.23	14.1	4.01	7.87	1.02	0.72	99.80
	4×	51.3	2.76	17.1	0.24	14.5	4.49	8.80	0.40	0.31	99.85
W-2	1×	52.1	0.88	15.5	0.16	10.8	6.71	10.90	2.22	0.58	99.77
	2×	52.0	1.03	16.2	0.16	10.8	6.52	11.10	1.63	0.47	99.81
	3×	51.4	1.10	16.3	0.17	10.8	6.71	11.21	1.59	0.41	99.69
	4×	51.4	1.14	16.6	0.16	10.9	6.78	11.40	1.12	0.31	99.81
GSP-2	1×	66.0	0.76	17.7	0.04	4.6	1.10	2.45	1.65	3.76	98.02
	2×	63.2	0.87	20.3	0.05	4.8	1.23	2.67	1.49	3.50	98.03
	3×	62.7	0.92	20.7	0.06	4.7	1.26	2.79	1.42	3.28	97.87
	4×	62.8	0.91	21.5	0.04	4.8	1.30	2.84	1.02	2.89	98.08
RGM-1	1×	73.0	0.28	14.6	0.04	1.9	0.28	1.25	3.55	4.05	98.94
	2×	71.9	0.31	15.8	0.05	1.9	0.33	1.38	2.86	3.62	98.12
	3×	69.7	0.35	18.5	0.05	2.0	0.34	1.58	2.54	3.32	98.39
	4×	67.9	0.36	20.4	0.05	2.2	0.40	1.77	2.33	3.23	98.61

The samples were fused between once and 4 times (1× - 4×). All data are given in wt.% of the oxides.

**Figure 5:**
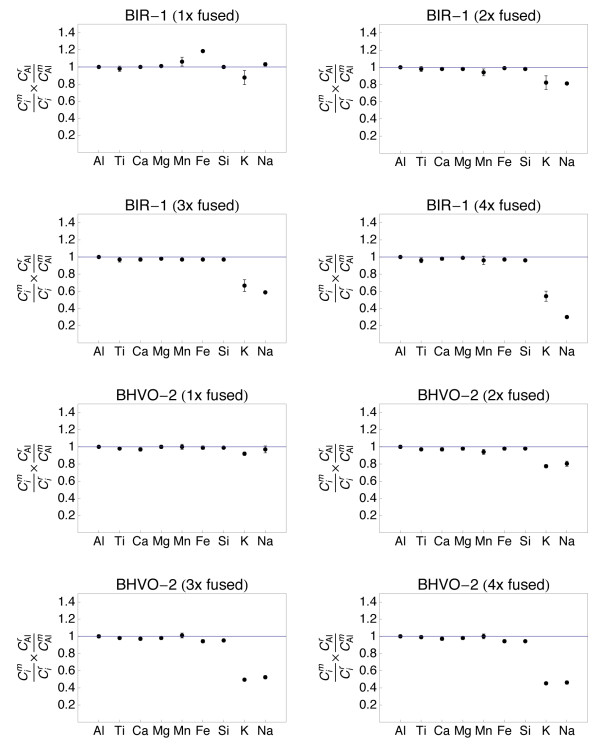
**Plots showing the major and minor element composition of quenched spheres (1 - 4 times fused) of basaltic standards BIR-1 and BHVO-2**. The element concentrations were normalized to the reference concentrations [38] and to Al (see text for details). The 1σ error bars are outlined.

**Figure 6:**
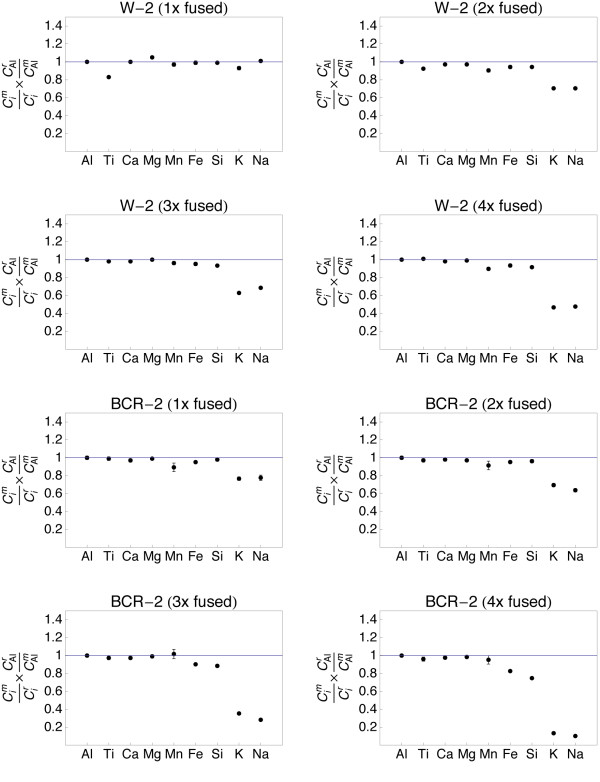
**Plots showing the major and minor element composition of quenched spheres (1 - 4 times fused) of standards W-2 and BCR-2**. The element concentrations were normalized to the reference concentrations [38] and to Al (see text for details). The 1σ error bars are outlined.

**Figure 7:**
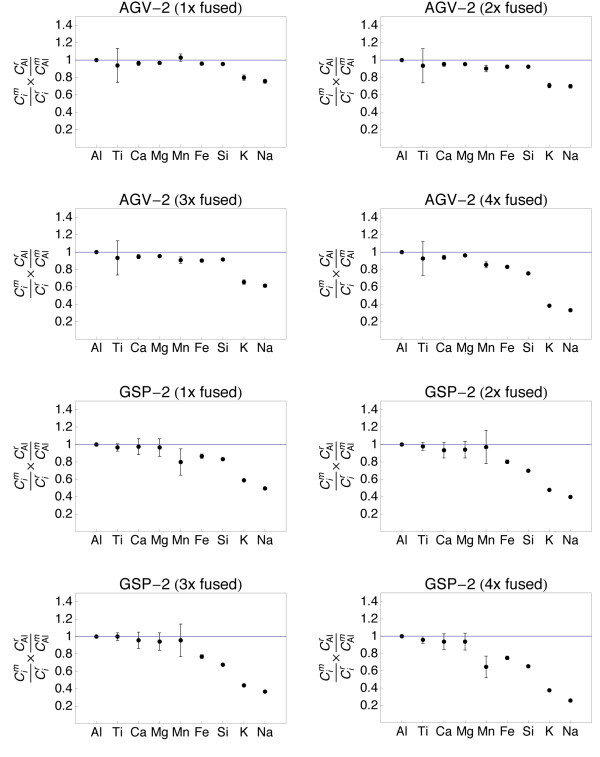
**Plots showing the major and minor element composition of quenched spheres (1 - 4 times fused) of andesite AGV-2 and granitoid GSP-2**. The element concentrations were normalized to the reference concentrations [38] and to Al (see text for details). The 1σ error bars are outlined.

**Figure 8:**
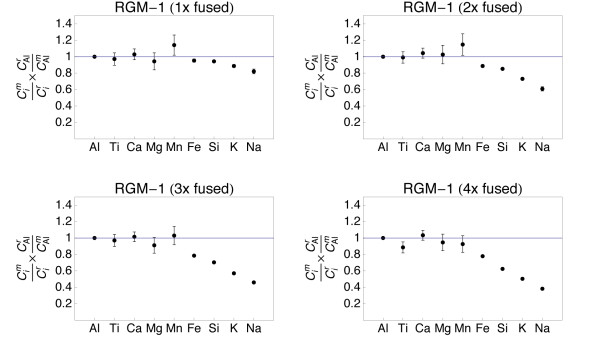
**Plots showing the major and minor element composition of quenched spheres (1 - 4 times fused) of rhyolite standard RGM-1**. The element concentrations were normalized to the reference concentrations [38] and to Al (see text for details). The 1σ error bars are outlined.

We have displayed all data with normalization on the concentration of the respective reference value of the element and on Al (Figs. 5, 6, 7 and 8). The measured to reference ratios were normalized to Al because this was the most refractory [i.e. has the highes evaporation temperature; [31,39]] major element in the samples and was not expected to be lost by evaporation. This is common practice when displaying chemical data with respect to volatility. A ratio of one indicates that the respective element was not lost during melting. A ratio <1 indicates evaporation. In such a case, the respective value gives the fraction of the element that remained in the sample. A ratio exceeding unity can only be due to sample heterogeneity.

The refractory element (Al, Ti, Ca, Mg) data from all samples demonstrate that homogeneity increases with the number of fusion steps. A single fusion resulted in significant deviation of e.g., Fe (BIR-1, Fig. 5) or Ti (W-2, Fig. 6) from the normalized reference concentrations. Subsequent fusion steps removed the heterogeneities. Increasing the number of fusion steps, however, also led to increasing loss of moderately volatile elements Na and K and, with more intense fusion, also Si and Fe (Figs. 5, 6, 7 and 8).

It is obvious from Figs 5, 6, 7 and 8 that the moderately volatile elements Na and K were lost from most samples, even when samples are levitated and fused only once. Exceptions were mafic samples BHVO-2 (Fig. 5) and W-2 (Fig. 6), which showed no loss of Na and K when fused once. All samples that were fused between 2 and 4 times show a loss of 20 - 90% of Na and K.

Samples, which have been 2 - 4 times levitation melted showed concentrations of Al, Ti, Ca and Mg that agreed within ± 5% with the respective reference values. Manganese, Fe and Si also agreed within ± 5% with the reference values, if samples were not fused more than 3 times. Four times of melting led to loss of alkalis, but also of up to 40% of Si from the felsic sample RGM-1 (Fig. 8). Loss of Mn, Fe and Si was less severe in the SiO_2_-poor, mafic materials.

**Figure 9:**
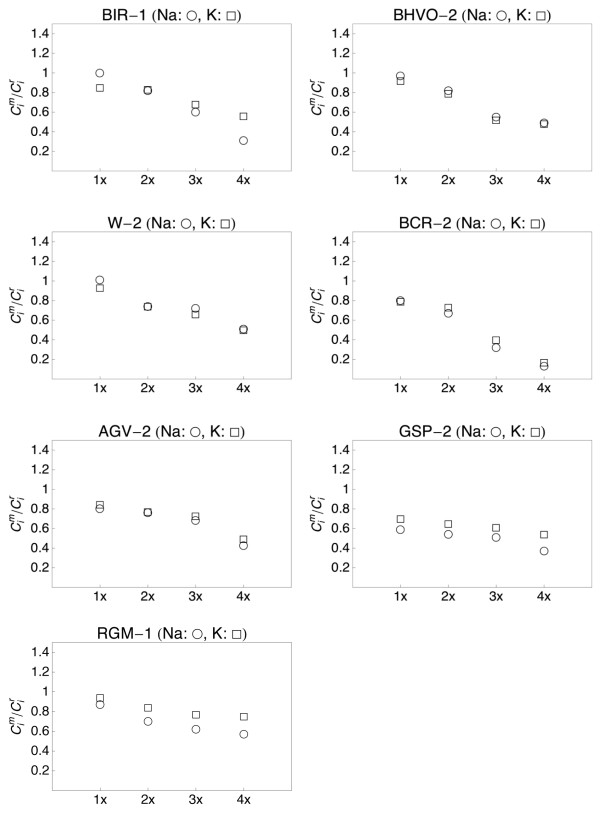
**Plot of Na (open circle) and K (open square) concentrations in 7 USGS rock standards normalized to the data reported for these materials in **[38]. The number of fusion events is indicated (1 - 4 times).

Loss of Na and K was clearly related to the number of fusion steps (Fig. 9). The loss of K correlated with the loss of Na in all, except the most SiO_2_-rich samples. In the most SiO_2_-rich samples GSP-2 and RGM-1 the relative loss of Na was systematically higher than the loss of K (Fig. 9).

### Alkali evaporation experiments

**Figure 10:**
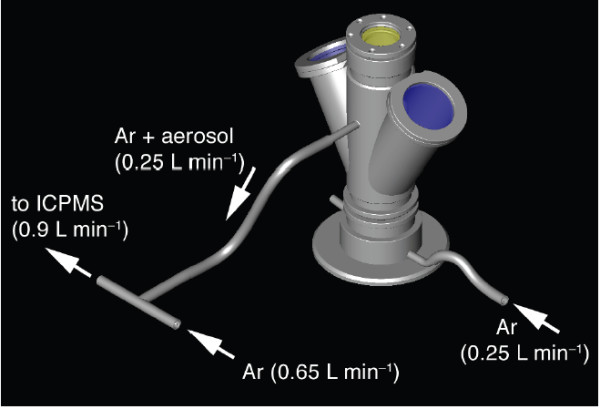
**Sketch illustrating the set up for the evaporation experiment**. The exhaust of the levitation chamber was connected to the inlet of the ICPMS. Argon was used as levitation gas.

Alkali (Na, K) evaporation experiments were conducted with a picritic basalt PB-63. The chemical composition of PB-63 was previously determined by X-ray fluorescence (XRF, Göttingen, Table 2). The liquidus temperature was 1620 °C. The picrite was pre-fused to a sphere in a graphite crucible before it was placed in the levitation nozzle. We used the closed sample chamber with Ar as levitation gas. The outlet of the levitation apparatus was directly connected to the source of an ICPMS in order to monitor the composition of the evaporated material (Fig. 10). The sample was heated and fused with variable laser energy. We monitored the stability of the sphere using the video system.

**Table 2:** Chemical composition of the starting materials.

	Picrite (PB-63, XRF*)	Lherzolite GZG1275/1 (XRF**)	Lherzolite GZG1275/1 (EPMA)	Lherzolite + metal oxides (mass balance)
SiO_2_	37.5	43.4	44.3	26.47
TiO_2_	0.63	0.45	0.49	0.27
Al_2_O_3_	6.10	7.2	7.3	4.40
MnO	0.2	0.13	0.09	0.08
FeO	13.9	7.7	7.4	39.63
CoO	0.02	n.a.	n.a.	0.14
NiO	0.15	n.a.	n.a.	3.95
MgO	26.7	33.1	34.1	20.18
CaO	4.00	5.9	6.0	3.59
Na_2_O	0.34	0.2	0.08	0.12
K_2_O	0.22	0.02	< 0.01	0.01
P_2_O_5_	0.06	0.01	< 0.01	< 0.01
H_2_O^+^	8.40	n.a.	n.a.	-
H_2_O^-^	0.78	n.a.	n.a.	-
Total	99.0	98.1	99.8	-
t_liquidus_***	1620°C	1655°C	1655°C	1240°C

Data are given in wt.% of the oxides.

*all Fe as FeO, **from Schoenberg et al., 2006., ***MELTS, water-free basis

The intensities of ^23^Na, ^24^Mg, ^27^Al, ^29^Si, ^39^K, ^43^Ca, ^55^Mn and ^57^Fe were continuously monitored with using a PERKIN ELMER DRC II quadrupole mass spectrometer. Dwell times of the isotopes were matched to achieve approximately the same intensities to minimize counting errors especially when determining element ratios.

The relative sensitivity factors (RSF_i_) for an isotope j of element i were calculated according to:

(1) RSFi=CiCNa×IN23aIij

The RSF values were determined by analyzing the aerosol that was released from the NIST 610 standard glass during ultra violet (UV, λ = 193 nm) excimer laser ablation (Table 3). In order to average out daily variations, we have adopted the mean RSF values that were obtained over a period of 8 month. Since aerosol from the UV laser ablation and from evaporation experiments will not necessarily have the same properties, concentration data from this study have a larger intrinsic error. It was, however, the purpose of this experiment to demonstrate a possible application of aerodynamic levitation rather than determining the exact composition of the evaporated material.

**Table 3:** List of the RSF values that were obtained in a period of 8 month for isotopes analyzed in this study.

Date	RSF_24 Mg_	RSF_27Al_	RSF_29Si_	RSF_39K_	RSF_43Ca_	RSF_55 Mn_	RSF_57Fe_
August 27, 2009	2.32	2.34	62.82	0.73	326.92	0.33	17.63
April 23, 2009	1.95	1.74	62.90	1.69	299.38	0.29	13.97
March 1, 2009	1.82	1.46	57.98	1.09	229.24	0.26	12.97
December 19, 2008	1.53	1.39	63.37	0.46	323.38	0.28	14.21
Average	1.91	1.73	61.77	0.99	294.73	0.29	14.70
Standarderror	0.16	0.22	1.27	0.27	22.67	0.02	1.02

The data were obtained by UV-laser ablation ICPMS of NIST 610 reference material.

We used Na as internal standard because we expected that the Na signal exceeded the signals of other elements in the evaporated aerosol. Sodium was the most volatile of the investigated elements [39]. The relative 1σ standard errors of the mean RSF values ranged from 2 to 27% with an average relative 1σ standard error of ± 10%.

The concentration C_i _of element *i *in the aerosol was calculated by means of:

(2) Ci=RSFi×IijIN23a×CNa

Equation 2 shows that the determination of *C_i _*requires knowing the concentration of the internal standard (i.e. Na). This was, a priori, not known for the aerosol that forms from the evaporated material. Except for oxygen, however, we monitored all major and minor elements (Na, Mg, Al, Si, K, Ca, Mn, Fe) that were present in the evaporated picrite PB-63 (Table 2) and assumed that the sum of the elements in the aerosol amounted 100 wt.%.

(3) $${\sum C}_{i}=100\;\text{wt}\text{.\%}$$

Combining Eq. 2 and Eq. 3 thus allowed determination of the composition of the aerosol without knowledge of the concentration of the internal standard element Na. This way, we acquired quantitative analyses of the evaporated material from the picrite PB-63 with high time resolution.

**Figure 11:**
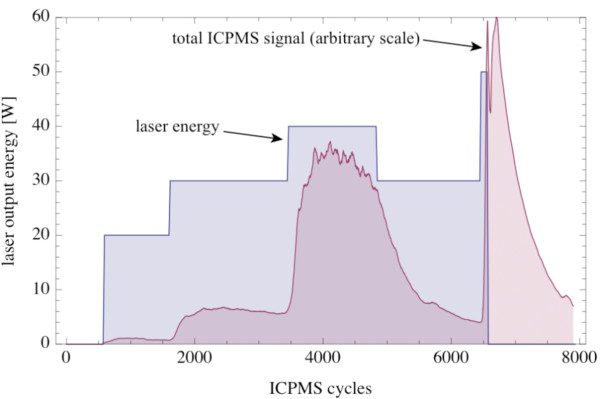
**Plot of the laser output energy vs. the number of ICPMS cycles (7.7 cycls s^-1^; blue)**. The red filled curve shows the normalized total ICPMS signal (Na, Mg, Al, Si, K, Ca, Mn and Fe; arbitrary scale). The total ICPMS signal was smoothed with a moving average of 20 cycles.

During the evaporation experiment, we heated the picrite PB-63 with laser energies between 20 and 50 W (Fig. 11). The sample was entirely molten during the whole experiment. The duration of the experiment was ~17 min. Evaporation started at 20 W laser energy, but became pronounced at 30 W (Fig. 11). The total signal increased with increasing laser energy and hence temperature of the melt (Fig. 11). The response time of the system with respect to an increase in laser energy was ~10 s. The signal exponentially decayed after switching off the laser with a half live of ~50 s (Fig. 11).

**Figure 12:**
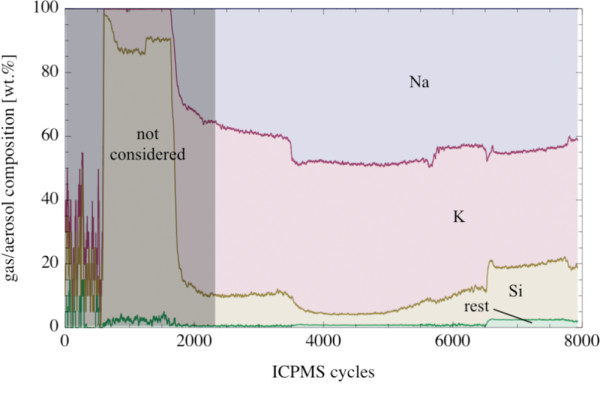
**Plot of the composition of the evaporated gas/aerosol from picrite PB-63 vs. number of cycles (7.7 cycl s^-1^)**. The signals were smoothed with a moving average of 20 cycles.

**Figure 13:**
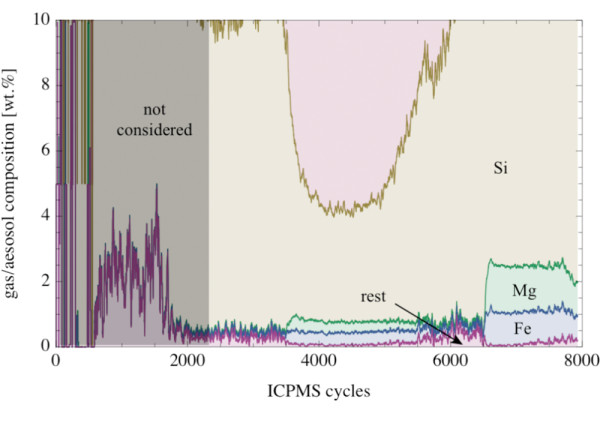
**Plot of the composition of the evaporated material from picrite PB-63 (enlarged part of Fig. 12)**. Minor components in the evaporated phase are Mg and Fe (~1 wt.% of the oxide, respectively) with the remaining elements Al, Ca and Mn amounting <0.5 wt.%. The signals were smoothed with a moving average of 20 cycles.

More than 80 wt.% of the aerosol was Na and K (Fig. 12). Both elements showed a strong correlation in the evaporated material. The Na/K-mass ratio in the evaporated material was in the range between 0.9 and 1.2. The amount of Si in the evaporated material varied between ~5 and ~18 wt.%. The Si-content increased with increasing temperature. Minor components in the evaporated material were Mg and Fe; both occurring in the 1 wt.% concentration level at the highest temperatures (Fig. 13). Other elements were only present as trace components in the evaporated material.

### Reduction experiments

The first reduction experiment was conducted with a mixture of natural spinel lherzolite powder (GZG1275/1, Table 2) that was mixed with 39 wt.% metal powder (88.6 wt.% Fe, 0.35 wt.% Co, 10.1 wt.% Ni). The metal mixture was prepared from analytical grade pure Fe, Co and Ni powders. The spinel lherzolite sample was provided by R. Schoenberg (Hannover) and was identical to the sample analyzed by Schoenberg et al., 2006 [40]. We have determined the chemical composition of the lherzolite by EPMA on a glassy sample that was homogenized by means of levitation melting (Table 2), results are identical within uncertainty. The liquidus temperature of the lherzolite was computed with MELTS [41,42] at 1655°C. The lherzolite metal mixture was oxidized in a muffle furnace (~1000°C, ~3 h) in air before melting and levitation in order to transfer the metal to oxides. The change in color of the powder from dark gray towards reddish brown suggested that metal was oxidized. The oxidized powder was re-ground and analyzed by means of X-ray powder diffraction using a Phillips PW 1710 with Cu-Kα radiation (5° < 2Θ < 70°, 0.02° steps, 0.5 s step^-1^). No Fe and Ni metal peaks were identified in the diffraction pattern. Instead, hematite (Fe_2_O_3_) and bunsenite (NiO) and olivine were the predominant phases in the pattern.

The liquidus temperature of the oxidized lherzolite metal mixture (see Table 2 for chemical composition) was determined with using MELTS [42] to be 1240°C with FeO-rich olivine as liquidus phase.

**Figure 14:**
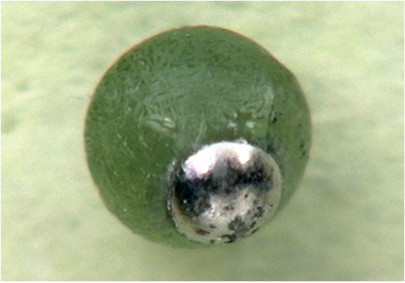
**Photography of the reduced lherzolite-metal oxide mixture**. Metal exsolved from the silicate. The diameter of the sphere was ~2 mm. The material was levitated with a mixture of 98 vol.% Ar and 2 vol.% H_2_.

The starting material was fused in a graphite crucible to a small sphere. The sphere was placed in the nozzle in the closed sample chamber in order to maintain a reducing atmosphere. The levitation gas was a commercially available mixture of 98 vol.% Ar and 2 vol.% H_2 _for ~10 min. The oxide-lherzolite mixture was levitated and melted for about 5 min in the reducing gas atmosphere. The experimental run product consisted of a silicate fraction and metal (Fig. 14). The metal sphere pointed towards the bottom of the levitation nozzle during melting. Both phases were liquid during the course of the experiment. The surface of the silicate sphere shows marks of skeletal olivine crystals that formed during quenching.

For the second reduction experiment, we used material of the NWA 869 L4-6 ordinary chondrite [43]. The chondrite powder was oxidized in air for ~24 h in order to transfer all metal into oxides. The sample was re-ground to powder in a hand mortar after oxidation. No metal grains were left. The sample was briefly pre-fused in a graphite crucible in air. The glass bead was then transferred into the levitation nozzle, where it was levitated using the 98 vol.% Ar + 2 vol% H_2 _gas mixture. The sample was fused for about 30 s in the reducing atmosphere and quenched by switching off the laser. It was embedded into resin for electron back scattered electron imaging and EPMA.

**Figure 15:**
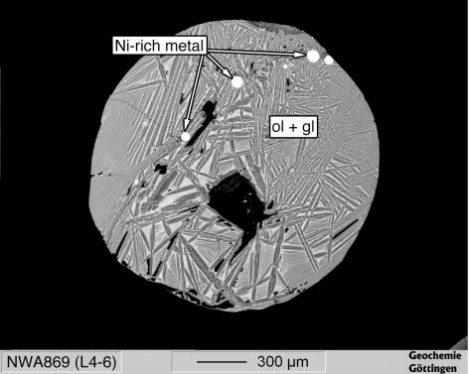
**Back scattered electron image of NWA 869 L4-6 ordinary chondrite**. The sample was entirely oxidized prior to levitation melting. A 98 vol.% Ar + 2 vol.% H_2 _mixture was used as levitating gas.

The run product consisted of quenched olivine crystals, interstitial glass and round metal droplets (Fig. 15). The composition of the silicate portion was determined by 10 randomly selected EPMA spots (10 μm spot diameter, Table 4). The FeO/SiO_2 _mass ratio of the silicate portion was 0.67. The NiO/SiO_2 _ratio of the silicate portion was 0.004. The metal blebs were rich in Ni with Ni contents between 29.5 and 59.8 wt.%. The concentration of Co was negatively correlated with Ni and varied between 0.69 and 1.38 wt.%. The concentrations of Si were <0.05 wt.% in all metal blebs. The mean metal composition is listed in Table 4.

**Table 4:** Result of EPMA measurements of silicate and metal of the reduced chondrite NWA 869.

Sample	NWA 869 (silicate)	NWA 869 (metal)
SiO_2_	40.0	-
TiO_2_	0.14	-
Al_2_O_3_	2.9	-
MnO	0.37	-
FeO	27.0	-
MgO	26.5	-
CaO	2.72	-
Na_2_O	0.09	-
K_2_O	< 0.05	-
P_2_O_5_	0.037	-
Cr_2_O_3_	0.59	-
NiO	0.15	-
Fe	-	53.1
Co	-	1.1
Ni	-	45.0
Total	100.4	99.2

Data are reported in wt.% of the oxides (silicate) or elements (metal).

## Discussion

### Bulk rock chemical analyses

The sample preparation protocol was time efficient (~5 min per sample) and required only 10 mg of sample powder. Testing different melting durations (Figs. 5, 6, 7 and 8) showed that 2 - 3 brief fusion steps (~5 s each) were the best compromise between sample homogeneity and loss of elements through evaporation. Non-volatile major and minor elements Si, Ti, Al, Ca, Mn and Fe could be determined with an uncertainty <5% relative (Table 2). Moderately volatile elements Na and K could not be determined with appreciable accuracy because they evaporate during sample melting (Fig. 9). The results demonstrated that levitation melting was a suitable preparation technique for bulk refractory elements, but not suited for moderately volatile elements like Na and K. The results on major and minor non-volatile elements support the conclusion by Pack et al. [28], Pack [29] and Patzer et al. [30] that aerodynamic levitation melting is a suitable method for the preparation of bulk rocks for analyses of refractory elements.

### Alkali evaporation experiments

The test with picrite PB-63 demonstrated that aerodynamic levitation in combination with ICPMS is a promising new technique for the investigation of evaporation from silicate melts. The experiment showed that Na and K evaporated in the same relative proportions from the melt. The Na/K mass ratio in the evaporated material (0.95 - 1.2) is similar to the ratio in the picrite PB-63 (Na/K = 1.22, Table 2). This observation was in agreement with the observed evaporative loss of Na and K from the USGS rock standards; except for the most silica rich samples (Fig. 9). This suggests that Na and K were similarly volatile under experimental conditions (1 bar, Ar-atmosphere). The most SiO_2_-rich rock samples (GSP-2, RGM-1) showed a preferred evaporation of Na relative to K (Fig. 9). The difference is likely related to a higher ratio of the alkali oxide activity coefficients $\gamma_{{\text{Na}}_{2}\text{O}}/\gamma_{{\text{K}}_{2}\text{O}}$ of felsic melts relative to mafic melts. An increase in γ leads to an increase in vapor pressure and hence elevated evaporation rates.

The response time to changes in laser energy were in the range of tens of seconds and allowed studies of evaporation processes with high time-resolution. Chondrules formed within a few minutes [34] in the solar nebula. Therefore, aerodynamic levitation in combination with ICPMS is a suitable technique to monitor evaporation of alkalis under different conditions (melt composition, atmosphere, temperatures). A smaller sample chamber may reduce the response times.

### Reduction experiments

The test with the lherzolite-oxide mixture demonstrated that siderophile elements could be extracted from silicate by means of levitation melting in reducing atmosphere. Siderophile trace elements can be extracted along with Fe and Ni by sample reduction. We demonstrated (Fig. 14) that levitation in an Ar-H_2 _mixture allows reduction of a major fraction of Fe and Ni to metal. Highly siderophile elements (e.g., platinum group elements) are expected to be quantitatively concentrated in the metal. Laser ablation ICPMS analyses of siderophile trace elements have been successfully conducted on iron meteorites [44,45].

The reduction experiment with the oxidized chondrite material showed that reduction of oxides (FeO, Fe_2_O_3_, NiO) to metal alloys occurred within 30 s. The high Ni concentration in the resultant metal was due to the low degree of reduction. Nickel is more siderophile than Co, which is more siderophile than Fe. It follows from the difference in redox potential that Ni is reduced first, followed by Co. The superchondritic Ni/Fe (0.85) and Co/Fe ratios (0.021) in the metal in the run product are clearly the result of the preferential reduction of Ni and Co. The chondritic ratios are 0.058 (Ni/Fe) and 0.0028 (Co/Fe) [39]. The Fe/Si ratio of the silicate is only little below the L-chondritic ratio. This demonstrates that most Fe remained in the silicate. The low Ni/Fe ratio in the silicate is evidence that most Ni was reduced to metal.

Chondrule formation took place in a more reduced environment of the solar nebula, which was essentially H_2, _and chondrule melting lasted over the range of minutes to tens of minutes. It is therefore plausible that metal in chondrites has formed by reduction during the brief chondrule melting event [36].

## Conclusions

Aerodynamic levitation in combination with microchemcial methods (EPMA, LA-ICPMS) is an efficient means of bulk rock preparation for analyses of non-volatile major, minor and trace elements. The strength of the technique lies in the absence of contamination and the option to analyze only small (~10 mg) samples. A further advantage is that no flux is used, which would dilute the elements of interest and could cause contamination, namely for trace and ultra trace elements. A disadvantage of the sample preparation technique involving containerless fusion is evaporative loss of moderately volatile elements like Na and K.

Aerodynamic levitation in combination with ICPMS online monitoring of the evaporated material allows time-resolved studies of the evaporation behavior at high temperatures. It is demonstrated that Na and K are similarly volatile at 1 bar Ar atmosphere. This observation is confirmed by results from fused rock standards.

Levitation and melting experiments in reduced atmosphere demonstrated that metal and silicate could be separated by reduction. The exsolution of metal did not affect the stability of the levitated melt. The experiment with an oxidized chondrite showed that Ni-rich metal is exsolved within ~30 s of melting.

## Competing interests

The authors declare that they have no competing interests.

## Authors' contributions

AP designed the levitation device and conducted first experiments. AP wrote the manuscript. KK and AK conducted the EPMA analyses. NA prepared the USGS samples. KS, NA and AP conducted the evaporation experiments and ICPMS analyses of the evaporated materials. All authors have read and approved the final manuscript.
